# Efficacy of Traditional Chinese Medicine, Maxingshigan-Weijing in the management of COVID-19 patients with severe acute respiratory syndrome: A structured summary of a study protocol for a randomized controlled trial

**DOI:** 10.1186/s13063-020-04970-3

**Published:** 2020-12-23

**Authors:** Congcong Zeng, Zhengzhong Yuan, Xiaoqiong Pan, Jizhou Zhang, Jiahui Zhu, Fan Zhou, Zhuocheng Shan, Ye Yuan, Ren Ye, Jinguo Cheng

**Affiliations:** 1 Department of Traditional Chinese Medicine, The First Affiliated Hospital of Wenzhou Medical University, Nanbaixiang, Ouhai District, Wenzhou, 325035 Zhejiang China; 2 Department of Traditional Chinese Medicine, The Second Affiliated Hospital and Yuying Children’s Hospital of Wenzhou Medical University, Lucheng District, Wenzhou, 325200 Zhejiang China; 3 Wenzhou Hospital of Traditional Chinese Medicine, Ouhai District, Wenzhou, 325035 Zhejiang China; 4 Wenzhou Medical University, Chashan Higher Education Park, Wenzhou, 325035 Zhejiang China; 5 Department of Traditional Chinese Medicine, Wenzhou Center Hospital, Lucheng District, Wenzhou, 325200 Zhejiang China

**Keywords:** COVID-19, Randomized controlled trial, protocol, Traditional Chinese medicine, Maxingshigan-weijing, Symptom

## Abstract

**Objectives:**

We aimed to test our expectation that additional administration of Traditional Chinese medicine (TCM), maxingshigan-weijing decoction, is more effective in the management of COVID-19 patients compared to those treated with routine supportive care alone.

**Trial design:**

This is a multicenter, open-label 2-arm (1:1 ratio) randomized controlled trial.

**Participants:**

Patients will be recruited from 3 hospitals in Wenzhou China: the First Affiliated Hospital of Wenzhou Medical University, the Second Affiliated Hospital of Wenzhou Medical University and Wenzhou Center Hospital. The inclusion and exclusion criteria are as follows:

*Inclusion criteria*

1. Participants are 18-85 years of age, either male or female.

2. Diagnosed as positive for severe acute respiratory syndrome coronavirus 2 (SARS-CoV-2)

3. Symptomatic. Mild (mild clinical symptoms without signs of pneumonia in chest X-ray) and Moderate (fever or respiratory symptom with signs of pneumonia in chest X-ray) .

1. Signed the informed consent before treatment.

2. Agreed not to enroll in any other clinical trials.

3. Inpatients

*Exclusion criteria*

1. < 18 or > 85 years old.

2. Pregnancy and lactation.

3. Serious heart, liver, kidney and hematopoietic system diseases, abnormal liver or kidney function.

4. Suffering from other known virus pneumonia.

5. Allergic to Chinese herbal medicine or suffering from allergies.

6. Critical patients (respiratory failure treated by mechanical ventilation or shock or multiple organ failure).

**Intervention and comparator:**

Patients in the control group will receive routine supportive clinically care including the therapies of anti-viral, anti-bacterial and ameliorating the related symptoms, while patients in TCM group will be asked to take maxingshigan-weijing decoction (composed of 14 Chinese herbal medicines), orally 200 mL 2 times daily, for 14 consecutive days in addition to routine supportive care as mentioned above.

Maxingshigan-weijing decoction consists of 10 g of Herba Ephedra (Mahuang), 10 g of Amygdalus Communis Vas (Xingren), 45 g of Gypsum Fibrosum (Shigao), 30 g of Rhizoma phragmitis (Lugen), 20 g of Peach kernel (Taoren), 20 g of Winter Melon kernel (Dongguaren), 30 g of Trichosanthes Kirilowii Maxim (Gualou), 12 g of Pericarpium Citri Reticulatae (Chenpi), 12 g of Rhizoma Pinelliae (Jiangbanxia), 12 g of caulis bambusae in taeniis (Zhuru), 30 g of semen lepidii (Tingliz), 15 g of semen lepidii (Shichangpu), 10 g of curcuma zedoary (ezhu) and 5 g of Radix Glycyrrhizae (Gancao).

**Main outcomes:**

The primary outcome will be the number of days until the clinical symptom of fever improves in the first 14 days of treatment following randomisation. Fever will be defined as an improvement when the temperature is less than 37°C.

Secondary outcomes will be TCM Syndrome Scores, the time it takes until individuals have negative test results for SARS-CoV-2 nucleic acid, the proportion of cases with chest X-ray improvements and the rate of symptom (fever, cough, malaise, shortness of breath) recovery.

TCM Syndrome Scoring System is a checklist covering 4 main, 7 secondary and 13 accompanying items. The 4 main items consisting of fever, cough, malaise and shortness of breath, use a four-point scale (0, 2, 4 and 6) depending on the severity; the 7 secondary items including dysphoria, diarrhea, pharyngalgia, expectoration, muscular soreness, nasal obstruction and rhinorrhoea use 0-3-point scale; the 13 accompanying items contain chest pain, headache, aversion to cold, dizziness, nausea and vomiting, anorexia, abdominal distension, dry mouth, anxiety, spontaneous sweating, insomnia, wheezing and blood tinged sputum, and each item is rated on 0-1 scale ( 0 stands for asymptomatic, 1 stands for symptomatic ). The total scores sum up to a range from 0 to 58, with higher scores indicating more severe levels of disease.

**Randomization:**

Minimization method will be used, balancing the two arms for pneumonia severity. Patients are randomized (1:1 ratio) to each group. Clinical researchers will get a random sequence number which is automatically generated by a random number generator (IBM Corp., Armonk, NY, USA), and sequentially number them in an opaque envelope. Researchers will open random allocation envelopes and assign participants accordingly. Eligible patients will be randomly divided into a routine supportive care group and a routine supportive care plus oral administration of traditional Chinese medicine group, with 70 patients in each group.

**Blinding (masking):**

This is an open-label study. The statistical analysis will be carried out by the Professor of Statistics at Wenzhou Medical University, who is blinded to patient allocation.

**Numbers to be randomised (sample size):**

The previous study reported the efficacy of TCM for COVID-19 and H1N1 influenza patients, the median survival time in the TCM group is estimated as 3 days; this time will be 1.5 times longer in the control group. Accordingly, Kaplan-Meier method and log-rank test will be used.

And assuming a statistical power of 70% (one-sided type-1 error of α = 5%, β = 30%) and a rate of withdrawal and loss to follow-up of 10%, we plan to include 140 participants in both groups ( TCM group = 70, control group = 70).

**Trial Status:**

The trial protocol is Version 2.0, October 14, 2020. Recruitment began March, 2020, and is anticipated to be completed by December 31, 2020.

**Trial registration:**

Chinese Clinical Trial Registry, ChiCTR2000030759.

Registered on 13 March 2020.

**Full protocol:**

The full protocol is attached as an additional file, accessible from the Trials website (Additional file 1). In the interest in expediting dissemination of this material, the familiar formatting has been eliminated; this Letter serves as a summary of the key elements of the full protocol.

**Supplementary Information:**

The online version contains supplementary material available at 10.1186/s13063-020-04970-3.

## Supplementary Information

**Additional file 1.** Full Study Protocol.

## Data Availability

The corresponding author has access to the final trial information, and the data will be available on reasonable request.

