# Supplementary material for: Efficacy of Traditional Chinese Medicine, Maxingshigan-Weijing in the management of COVID-19 patients with severe acute respiratory syndrome: A structured summary of a study protocol for a randomized controlled trial
Source: Trials. 2020 Dec 23;21:1029. doi: 10.1186/s13063-020-04970-3 (PMC7755980; doi:10.1186/s13063-020-04970-3)
Supplement: Supplementary file 1 — Additional file 1. Full Study Protocol. [file 13063_2020_4970_MOESM1_ESM.doc]

**The efficacy and safety of Traditional Chinese Medicine, Maxingshigan-Weijing in the management of COVID-2019 patients: An open-label multicenter, randomized controlled trial**

Congcong Zeng^a,1^, Zhengzhong Yuan^a,1^, Xiaoqiong Pan^b^, Jizhou Zhang^c^, Jiahui Zhu^d^, Fan Zhou^e^, Zhuocheng Shan^a^, Ye Yuan^d^, Ren Ye^a*^ and Jinguo Cheng^d*^

Corresponding authors: Ren Ye and Jinguo Cheng

Address: Wenzhou Medical University, Chashan Higher Education Park, Ouhai District, 325035, Wenzhou, Zhejiang, China.

Email address:

Congcong Zeng:zengcongcong1990@126.com

Zhengzhong Yuan:wzyzz2008@126.com

Ren Ye:yeren601@163.com. Tel.: 13706660307

Jinguo Cheng: wzwsjcjg@163.com. Tel.: 13857797188

^1^These authors equally contributed to this work.

Full list of author information is available at the end of the article.

**Abstract**

**Introduction:** The outbreak of a novel coronavirus (SARS-CoV-2) and associated COVID-19 disease in late December 2019 has led to a global pandemic. It directly leads to high morbidity and mortality, but also results in a devastating effect on the global economy. Nowadays many trials are underway on this disease to evaluate the efficacy of various therapeutic remedies. This study aims at investigating the effect of Traditional Chinese Medicine, maxingshigan-weijing for treating patients with COVID-19.

**Methods and analysis:** This is an open-label, multicenter randomized controlled clinical trial. One hundred and forty patients infected by SARS-CoV-2 will be randomly assigned to the control group or the experimental group ( Traditional Chinese Medicine, TCM group ) in an equal ratio. The patients in control group will receive routine supportive clinically care including the therapies of anti-viral, anti-bacterial and ameliorating the related symptoms, while patients in TCM group will be asked to take traditional Chinese medicine, maxingshigan-weijing for consecutive 14 days in addition to supportive care. All data will be gathered at baseline and on days 3, 7, 10 and 14. The primary outcome measure will be the number of days till at least one of the clinical symptoms (fever, cough, malaise, shortness of breath) improves in the first 14 days of treatment. Secondary outcome measures will include TCM Syndrome Scoring System and the time of negative of SARS-CoV-2 nucleic acid. A laboratory test will be taken before and after treatment to assess the safety of TCM.

**Discussion:** The study may help to identify the the efficacy and safety of Traditional Chinese Medicine, Maxingshigan-weijing in treating COVID-2019.

**Trial registration:** Chinese Clinical Trial Registry, ChiCTR2000030759. Registered on March 13^th^ 2020, <http://www.chictr.org.cn/.>

**Keywords:** SARS-CoV-2, COVID-2019, Traditional Chinese Medicine ( TCM ), Maxingshigan-weijing decoction, Randomized controlled trial, Protocol

**Background**

In December 2019, an unexplained pneumonia broke out in Wuhan City, Hubei Province. And it was identified and named COVID-2019 (Corona Virus Disease-2019) by the World Health Organization ( WHO ) on 30^th^ January 2020[1]. Unfortunately, this virus quickly spread throughout China[2] and had been identified as a pandemic and a health emergency of global concern by WHO[3]. What is even more frightening is that the virus has spread with astonishing speed around the world, many people in Europe and United States have suffered from COVID-2019[4].

SARS-CoV-2 is the causative pathogen of COVID-19, identified as the seventh type of coronavirus to infect humans [5]. Six other kinds of coronaviruses are known to cause human disease, including severe acute respiratory syndrome coronavirus (SARS-CoV) and Middle East respiratory syndrome coronavirus (MERS-CoV) which had caused two large-scale pandemics in the past two decades [6,7]. According to the genome characteristics, coronavirus is separated into four genera: α-CoV, β-CoV, γ-CoV, and δ-CoV [8]. Deep sequencing revealed that this novel coronavirus shares 79.6% sequence identity with SARS-CoV and belongs to β-CoV [9].

In the past two decades, human fought with SARS-CoV and MERS-CoV hardly, they firstly and fastly used those broad-spectrum antiviral drugs, including interferons, ribavirin, and cyclophilin inhibitors, in addition to supportive care[10,11,12]. The second category of candidate antivirals clinicians chose, through screening of chemical libraries that comprise large numbers of existing drugs or databases that contain information on transcriptional signatures in different cell line, consisted of mycophenolic acid, lopinavir, chloroquine and so on[13,14]. Furthermore, widely usage of Traditional Chinese Medicine (TCM) to treat these two infectious diseases was another option for Chinese doctors, especially in the treatment of patients infected with SARS-CoV in 2002-2003[15]. It was reported that the patients with SARS-CoV infection had benefited from TCM treatment, including amelioration of side effect of conventional therapeutics[16,17,18].

Faced with COVID-2019, because of no clinically specific drugs currently[19], doctors choose the drugs mentioned above empirically but the clinical efficacy is unsatisfactory and uncertain. During these challenging and unprecedented times, the current clinical guideline in China suggest both conventional medicine and TCM should be used for the treatment of patients with infection of SARS-CoV-2[20].

Based on these evidence and experience, there is a general expectation that TCM would be a valuable weapon in the armory against SARS-CoV-2. So an open-label, multicenter randomized controlled study will be carried out to evaluate the efficacy and safety of Traditional Chinese Medicine in treating COVID-2019. This study is conducted in Wenzhou city, Zhejiang Province, because Wenzhou is one of the worst-hit cities outside hubei province by COVID-2019.

**METHODS**

**Study design**

This is an open-label, multicenter, randomized controlled, comprehensive treatment clinical study, with outcome assessors and data analysts blinded to the group assignment. Eligible participants with Mild and Moderate Pneumonia caused by SARS-CoV-2 will be randomly assigned to either the experimental ( Traditional Chinese Medicine + Routine supportive care ) or the control group ( Routine supportive care ) in an equal proportion. Routine supportive care remains the major treatment, including the therapies of anti-viral, anti-bacterial and ameliorating the related symptoms[21]. The study consisted of three phases: a screening period, a treatment period of 14 days, and a follow-up period of 2 weeks. Fig. 1 briefly shows the study flow chart, and Fig. 2 enumerates the treatment schedule and outcome measures. The study adheres to the Standard Protocol Items: Recommendations for Intervention Trials (SPIRIT 2013) checklist [22].

**Participants and Recruitment**

Participants will be recruited from 3 hospitals, the First Affiliated Hospital of Wenzhou Medical University, the Second Affiliated Hospital of Wenzhou Medical University and Wenzhou Center Hospital. These 3 hospitals are appointed to treat patients with COVID-19. To recruit target subjects, the study researchers will send a message to every inpatient when they are diagnosed with SARS-CoV-2. If the inpatients are eligible and interested in taking part in the study, they will be fully informed of the project information including the study procedures, potential benefits and risks. Written informed consent includes provisions for use of study data and biological specimens in future researches will be obtained from the patient or their substitute decision-makers before the allocation. All participant have the right to withdraw from the study at any time.

**Inclusion criteria**

1. Participants are 18-85 years of age, either male or female.
2. Diagnosed as positive for severe acute respiratory syndrome coronavirus 2 (SARS-CoV-2)
3. Symptomatic. Mild (mild clinical symptoms without signs of pneumonia in chest X-ray), and Moderate (fever or [respiratory](../../../../C:/Users/zcc/Downloads/%25E6%259C%2589%25E9%2581%2593/Dict/8.9.0.0/resultui/html/index.html#/javascript:;) [symptom](../../../../C:/Users/zcc/Downloads/%25E6%259C%2589%25E9%2581%2593/Dict/8.9.0.0/resultui/html/index.html#/javascript:;) with signs of pneumonia in chest X-ray) .
4. Signed the informed consent before treatment.
5. Agreed not to enroll in any other clinical trials.
6. Inpatients

**Exclusion criteria**

1. < 18 or > 85 years old.
2. Pregnancy and lactation.
3. Serious heart, liver, kidney and hematopoietic system diseases, abnormal liver or kidney function.
4. Suffering from other known virus pneumonia.
5. Allergic to Chinese herbal medicine or suffering from allergies.
6. Critical patients ( respiratory failure treated by mechanical ventilation or shock or multiple organ failure).

**Criteria for discontinuing**

1. The participant decides to discontinue the intervention, the trial or data collection at any time for any reason.
2. Non-compliant patient.
3. In the case of the onset of serious adverse events.
4. The aggravating pathology changes which require ventilator support for treatment during the intervention.

Participants discontinued the trial will be recorded throughout the intervention and followup periods, their data will be collected in the case report form. If the trial wouldn’t continue due to serious adverse events or aggravated illness, appropriate treatment will be taken.

**Sample size**

Based on previous clinical practice of using TCM fought against SARS and H1N1 influenza patients [23], it is estimated the median survival time in the TCM group as 3 days, and 1.5 times longer in the control group. Concerning the analysis of the primary endpoint, the duration of time before improvement of at least one of the common cold-like symptoms (fever, cough, malaise, shortness of breath) , will be estimated using the Kaplan-Meier method, and

the survival curves will be compared between groups using the log-rank test. And this study is designed to detect a statistically significant difference with a level of 5% and a power of 70% (one-sided type-1 error of
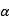
 =5%，
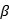
 =30%), so approximately 61 participants in each group will be needed. Thus, under the prediction of a dropout rate of 10%, the sample size of 140 participants will be sufficient to detect a clinically important difference in this trial.

**Randomization**

Minimization method will be used, with balancing of the arms with pneumonia severity. Patients are randomized (1:1 ratio) to each group. Clinical researchers will get a random sequence number which is automatically generated by a random number generator (IBM Corp., Armonk, NY, USA), and sequentially number them in an opaque envelope. Researchers will open random allocation envelopes and assign participants accordingly. Eligible patients will be randomly divided into a routine supportive care group and a routine supportive care plus oral administration of traditional Chinese medicine group, with 70 patients in each group.

**Blinding**

Because of the odor and color of traditional Chinese medicine decoction, the patients and clinical therapists will be unblinded in this trial. But the outcome assessors and data analysts will be blinded throughout the entire study.

**Prohibition and permission for concomitant treatment**

All concomitant care and interventions are permitted other than concomitant receipt of any other experimental treatment.

**Patient and public involvement**

Patients in this study will not be involved in the design, recruitment or conduction of the study, but they will be informed of the Clinical examination results they had accepted.

**INTERVENTIONS**

Maxingshigan-weijing decoction consists of 10 g of Herba Ephedra (Mahuang), 10 g of Amygdalus Communis Vas (Xingren), 45 g of Gypsum Fibrosum (Shigao), 30 g of Rhizoma phragmitis (Lugen), 20 g of Peach kernel (Taoren), 20 g of Winter Melon kernel (Dongguaren), 30 g of Trichosanthes Kirilowii Maxim (Gualou), 12 g of Pericarpium Citri Reticulatae (Chenpi), 12 g of Rhizoma Pinelliae (Jiangbanxia), 12 g of caulis bambusae in taeniis (Zhuru), 30 g of semen lepidii (Tingliz), 15 g of semen lepidii (Shichangpu), 10 g of curcuma zedoary (ezhu) and 5 g of Radix Glycyrrhizae (Gancao). Maxingshigan-weijing decoction will be prepared by TCM pharmacy of the First Affiliated Hospital of Wenzhou Medical University.

Inpatients will be randomly assigned to two groups receiving either Routine supportive care alone which includes Stay in bed, Nasal cannula of oxygen therapy, broad-spectum antibiotics and antivirals, or the combination of Maxingshigan-weijing decoction for continuous 14 days, 200 mL twice a day at 10 AM and 4PM. All participants will be hospitalized so that they could be quarantined and closely observed. What is more, to ensure compliance with the medication, patients will be asked to return the empty wrapping papers. If there will be any remaining medicine, it will be recorded.

**MEASUREMENTS**

***Primary Outcome***

The primary outcome will be the number of days until the clinical symptom of fever improves in the first 14 days of treatment following randomisation. Fever will be defined as an improvement when the temperature is less than 37℃.

***Secondary outcomes***

**TCM Syndrome Scoring System**

### TCM Syndrome Scoring System will be used at the baseline and day 3, day 7, day 10 and day 14 of the drug intervention. It is a checklist covering 4 main, 7 secondary and 13 accompanying items. These items are the symptoms and signs summarized by experts according to the clinical manifestations of COVID-2019[24]. The main items consisting of fever, coughing, fatigue and short breath use a four-point scale (0, 2, 4 and 6) depending on the severity; the 7 secondary items including dysphoria, diarrhea, pharyngalgia, expectoration, muscular soreness, nasal obstruction and rhinorrhoea use 0-3-point scale; the 13 accompanying items contain chest pain, headache, aversion to cold, dizziness, nausea and vomiting, anorexia, abdominal distension, dry mouth, anxiety, spontaneous sweating, insomnia, wheezing and blood tinged sputum, and each item is rated on 0-1 scale ( 0 stands for asymptomatic, 1 stands for symptomatic ). The total scores sum up to a range from 0 to 58, with higher scores indicating more severe levels of disease.

**Coronavirus nucleic acid test**

The coronavirus nucleic acid test by reverse transcription polymerase chain reaction ( RT-PCR ) will be conducted at the baseline and day 3, day 7, day 10 and day 14 of the study intervention. The time ( in days ) of the respiratory tract sample and blood specimens SARS-CoV2 RT-PCR to be negative for two consecutive times based on the first testing time will be defined as primary outcome.

**Improvements of chest X-ray (CRX)**

An experienced radiologist who was blinded to the study allocation reviewed the chest computed tomography (CT) images from all patients. An improvement in CRX images was defined as a decreased area of infiltration, a decreased area of any radiologic abnormality, or decreased density of the ground-glass opacity or nodules.

**Rate of symptom recovery**

The rate of symptom (fever, cough, malaise, shortness of breath) recovery will be one of the secondary endpoints. Fever denoted the subaxillary temperature being 37 degrees or greater. The cough and malaise was self-reported by the patients. Recovery of symptoms will be defined as the complete resolution of fever, cough, malaise, shortness of breath.

***Safety outcomes***

Test safety will be monitored during this trial. Participants will accept a routine physical examination, including breath rate, heart rate, temperature, blood pressure, weight, routine urine test, routine blood test, liver, kidney function tests, and an electrocardiogram.

***Adverse event reporting and treating***

Adverse events (AEs) will be defined as unpredictable, undesirable symptoms, signs or diseases related to the administration of maxingshigan-weijing decoction and will be recorded and managed by the researchers during the treatment period. The researchers will scrupulously evaluate the relationship between these adverse events and the experimental drugs, then manage them quickly and properly. Furthermore, patients will be continuously followed up until their condition returns to normal. Participants who might be injured during the study will get a financial compensation.

***Quality control, data collection and management***

Trained research staff collect trial data carefully according to a standard protocol and complete paper case report forms (CRFs) accurately, completely, timely and reliably.

To keep the information confidential, the experimental data including primary and secondary outcomes will be carefully recorded in CRFs which will be identified by IDs rather than identifiable information and will be stored separately and securely in the linking document. To guarantee the data quality, the completed CRFs will be input in Microsoft Access by two researchers. For some emergent reasons, investigators were bound to

During the study, the First Affiliated Hospital of Wenzhou Medical University is responsible for making regular visits (once a week) to review trial conduct, and the Ethics Committee will monitor for protocol violations weekly, there was no conflict of interest with the sponsors or researchers.

**STATISTICAL ANALYSIS**

We will use log-rank test for the primary outcome, if the influence of confounding factors needs to be considered, Cox proportional hazard model can be used. For all secondary outcomes, Wilcoxon rank sum test will be used. For safety evaluation of TCM, two-sided t-test will be applied to each safety measurement after data normalization.

To better understand the nature the missing mechanisms, we will characterize the type of subjects who have missing data, then statistical models to impute missing values if the assumption of missing at random is met will be used.

**DISCUSSION**

Traditional Chinese Medicine has a long history of more than five thousand years, and has accumulated a lot of experiences in treating infectious diseases. It is widely popular in China and some Asia countries because of its good clinical therapeutic effect and fewer side effects is highly valued by the government of China.

Application of TCM in the treatment of SARS-CoV-2 is mainly inspired by the treatment of SARS in the late of 2002 in the Guangdong Province of China[25,26,27]. So in this campaign to contain and eradiate SARS-CoV-2, China government strongly suggest that TCM should be used in combination with conventional medicine in the treatment of COVID-19 patients. Therefore, those standard and strict controlled clinical studies are urgently needed to evaluate the efficacy of TCM in treating COVID-19. At the same time, fighting against current epidemics also provide an opportunity to test the true value of TCM in treating emerging contagious diseases.

In conclusion, it is meaningful and worthy to design this open-label, multicenter randomized controlled trial to test efficacy and safety of TCM in COVID-2019. We hope it could provide complementary and alternative treatments for all mankind in the battle with COVID-19.

**Ethics approval** **and Results dissemination**

The study has been approved by the Ethical Research Committees of the First Affiliated Hospital of Wenzhou Medical University (2020003). Written informed consent will be obtained from all participants by the investigator. And On the consent form, participants will be asked if they agree to use of their data should they choose to withdraw from the trial. Participants will also be asked for permission for the research team to share relevant data with people from regulatory authorities.

The trial results will be disseminated to participants, healthcare professionals, the public, and other relevant groups via Chinese Clinical Trial Registry ( [http://www.chictr.org.cn](http://www.chictr.org.cn/)/ ).

**Trial status**

The trial protocol is Version 2.0, October 14, 2020. Recruitment began March, 2020, and is anticipated to be completed by December 31, 2020.

**Consent for publication**

Not Applicable.

**Availability of data and materials**

The datasets used or analyzed during the current study will be available from the corresponding author upon reasonable request.

**Competing interests**

The authors declare that they have no competing interests.

**Funding**

This study is supported by the Wenzhou Municipal Science and Technology Bureau (CN) (ZY202003). The funder had no role in the design of the study; analysis, collection, and interpretation of the data; or the writing and decision for publication of the manuscript.

**Author Contributions**

CJG and YR are the principal investigators and corresponding authors in charge of prescribing Traditional Chinese medicine. ZCC and YZZ are the first authors responsible for preparing the manuscript. ZCC is the research investigator. YZZ is the second investigator. ZJH, ZF and SZC are the outcome assessors. CJG and YR conceived this trial and participated in its design. ZJZ and YY are responsible for data management and analysis. PXQ, ZCC and YZZ will coordinate the trial and contribute to the screening of patients, and are also involved in the recruitment of participants from clinics. ZCC will collate the data and prepared the manuscript. YZZ provided critical revision of the manuscript. All named authors adhere to the authorship guidelines of Trials. All authors have agreed to publication. Conceptualization: CJG and YR. Formal analysis: a statistician from the Clinical Evaluation and Analysis Centre of the First Affiliated Hospital of Wenzhou Medical University. Investigation: PXQ, ZCC and YZZ. Methodology: ZJH, ZF and SZC, ZJZ and YY. Supervision: CJG and YR. Writing original draft: ZCC. Writing review and editing: YZZ.

**Authors’ information**

a Department of Traditional Chinese Medicine, The First Affiliated Hospital of Wenzhou Medical University, Nanbaixiang, Ouhai District, 325035, Wenzhou, Zhejiang, China. b Department of Traditional Chinese Medicine, The Second Affiliated Hospital and Yuying Children’s Hospital of Wenzhou Medical University. c Wenzhou Hospital of Traditional Chinese Medicine. d Wenzhou Medical University, Chashan Higher Education Park, 325035, Wenzhou, Zhejiang, China. e Department of Traditional Chinese Medicine, Wenzhou Center Hospital.

**Acknowledgements**

We would like to thank Wenzong Zhang, Gang Liu and Xiaoqu Zhu (Wenzhou Hospital of Traditional Chinese Medicine) and Jun Yang (Wenzhou Center Hospital) for providing medical help.

**References**

1. Zhu N, Zhang D, Wang W, et.al. A novel Coronavirus from Patients with Pneumonia in China, 2019. N Engl J Med. 2020; 382(8): 727–733.

2. Hui DS, E IA, Madani TA, et al.The continuing 2019-nCoV epidemic threat of novel coronaviruses to globalhealth - The latest 2019 novel coronavirus outbreak in Wuhan, China. Int JInfect Dis. 2020;91:264-6.

3. World Health Organization. WHO Director- General’s remarks at the media briefing on 2019-nCoV on 11 February 2020. https://www. who.int/dg/speeches/detail/who-director-general-s-remarks-at-the-media-briefing-on-2019-nCov on-11-february-2020. Accessed February 19, 2020.

4. Coronavirus 2019‐nCoV, CSSE. Coronavirus 2019‐nCoV Global Cases by Johns Hopkins CSSE. (Available from: [https://gisanddata.maps.arcgis.com/apps/opsdashboard/index.html#/bda7594740fd40299423467b48e9ecf6](https://gisanddata.maps.arcgis.com/apps/opsdashboard/index.html#/bda7594740fd40299423467b48e9ecf6))

5. Min Zhou, Xinxin Zhang, Jieming Qu. Coronavirus disease 2019 (COVID-19): a clinical update. Front Med.DOI:10.1007/s11684-020-0767-8

6. Drosten, C. et al. Identification of a novel coronavirus in patients with severe acute respiratory syndrome. N. Engl. J. Med. 348, 1967–1976 (2003).

7. Zaki, A. M., van Boheemen, S., Bestebroer, T. M., Osterhaus, A. D. M. E. & Fouchier, R. A. M. Isolation of a novel coronavirus from a man with pneumonia in Saudi Arabia. N. Engl. J. Med. 367, 1814–1820 (2012).

8.Su S,Wong G,Shi W,Liu J, et.al. Epidemiology, genetic recombination, and pathogenesis of coronaviruses. Trends Microbiol 2016; 24(6): 490–502

9. [Peng Zhou](https://www.nature.com/articles/s41586-020-2012-7#auth-1), [Xing-Lou Yang](https://www.nature.com/articles/s41586-020-2012-7#auth-2), [Xian-Guang Wang](https://www.nature.com/articles/s41586-020-2012-7#auth-3), [Zheng-Li Shi](https://www.nature.com/articles/s41586-020-2012-7#auth-29), et al. A pneumonia outbreak associated with a new coronavirus of probable bat origin. Nature. 570, 270-273 (2020).

10. de Wilde AH, Raj VS, Oudshoorn D, Bestebroer TM, van Nieuwkoop S, Limpens RW, Posthuma CC, van der Meer Y, Barcena M, Haagmans BL, Snijder EJ, van den Hoogen BG. 2013. MERS-coronavirus replication induces severe in vitro cytopathology and is strongly inhibited by cyclosporin A or interferon-alpha treatment. J Gen Virol 94:1749–1760. doi:10.1099/vir.0.052910-0.

11. Frausto SD, Lee E, Tang H. 2013. Cyclophilins as modulators of viral replication. Viruses 5:1684–1701. doi:10.3390/v5071684.

12. Falzarano D, de Wit E, Martellaro C, Callison J, Munster VJ, Feldmann H. 2013. Inhibition of novel beta coronavirus replication by a combination of interferon-alpha2b and ribavirin. Sci Rep 3:1686. doi:10.1038/srep01686.

13. Barnard DL, Day CW, Bailey K, Heiner M, Montgomery R, Lauridsen L, Chan PK, Sidwell RW. 2006. Evaluation of immunomodulators, interferons and known in vitro SARS-coV inhibitors for inhibition of SARS-coV replication in BALB/c mice. Antivir Chem Chemother 17:275–284.

14. de Wilde AH, Jochmans D, Posthuma CC, Zevenhoven-Dobbe JC, van Nieuwkoop S, Bestebroer TM, van den Hoogen BG, Neyts J, Snijder EJ. 2014. Screening of an FDA-approved compound library identifies four small-molecule inhibitors of Middle East respiratory syndrome coronavirus replication in cell culture. Antimicrob Agents Chemother 58:4875–4884. doi:10.1128/AAC.03011-14.

15. Leung PC. The efficacy of Chinese medicine for SARS: a review of Chinese publications after the crisis. Am J Chin Med. 2007;35:575–81.

16. Tong X, Li A, Zhang Z, Duan J, Chen X, Hua C. et al. TCM treatment of infectious atypical pneumonia-a report of 16 cases. J Tradit Chin Med. 2004;24:266–9.

17. Liu X, Zhang M, He L, Li Y. Chinese herbs combined with Western medicine for severe acute respiratory syndrome (SARS) Cochrane Database Syst Rev. 2012;10:Cd004882.

18. Zhang MM, Liu XM, He L. Effect of integrated traditional Chinese and Western medicine on SARS: a review of clinical evidence. World J Gastroenterol. 2004;10:3500–5.

19. Luo H, Tang QL, Shang YX, Liang SB, Yang M, Robinson N, Can Chinese Medicine Be Used for Prevention of Corona Virus Disease 2019 (COVID-19)? A Review of Historical Classics, Research Evidence and Current Prevention Programs. Chin J Integr Med. 2020.

20. Li G, Clercq ED. Therapeutic options for the 2019 novel coronavirus (2019-nCoV) Nat Rev Drug Discov. 2020.

21. National Health Commission of the People’s Republic of China. Guideline for the diagnosis and treatment of COVID-19 infections (version 1–7). 2020. http://www.nhc.gov.cn/yzygj/zcwj2/ new_zcwj.shtml (accessed March 9, 2020)

22. Chan AW, et.al.SPIRIT 2013 Statement: Defining Standard Protocol Items for Clinical Trials. Ann Intern Med. 2013, 158(3):200-7.

23. Ming Le. Statistical analysis of clinical efficacy of integrated

traditional Chinese and western medicine in the treatment of SARS.

Journal of practical internal medicine of traditional Chinese

medicine,2005.19(6):529.

1. Qing Miao, Xiaodong Cong, Bing Wang et. al. Recognition and

reflection of TCM on pneumonia caused by novel

coronavirus.2020,61(2):1-4.

1. Zhong N, May RM, McLean AR, Pattison J, Weiss RA. Management and prevention of SARS in China. Philos Trans R Soc Lond B Biol Sci. 2004; 359:1115-6. e0146197.
2. JSM P, D P, Yuen KY ea. The Severe Acute Respiratory Syndrome. New Engl J 71. Med.. 2003; 249: 2431-41.
3. Jr TMF, Tsang KWT. Severe Acute Respiratory Syndrome. Nat Med. 2005; 4:95-106.


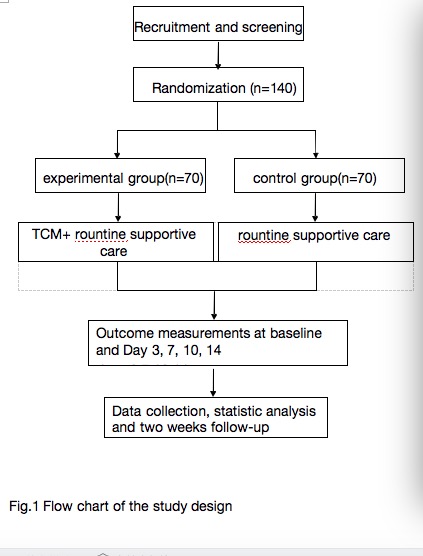


|  | **Pre** | **Research Phase** | | | | | | | | | | | | | | **Follow-up** | |
| --- | --- | --- | --- | --- | --- | --- | --- | --- | --- | --- | --- | --- | --- | --- | --- | --- | --- |
| **Time point** | **D0** | **D1** | **D2** | **D3** | **D4** | **D5** | **D6** | **D7** | **D8** | **D9** | **D10** | **D11** | **D12** | **D13** | **D14** | **D7** | **D14** |
| **Research activities** |  |  |  |  |  |  |  |  |  |  |  |  |  |  |  |  |  |
| Enrollment | × |  |  |  |  |  |  |  |  |  |  |  |  |  |  |  |  |
| Eligibility screen | × |  |  |  |  |  |  |  |  |  |  |  |  |  |  |  |  |
| Informed consent | × |  |  |  |  |  |  |  |  |  |  |  |  |  |  |  |  |
| Randomization | × |  |  |  |  |  |  |  |  |  |  |  |  |  |  |  |  |
| **Intervention activities** |  |  |  |  |  |  |  |  |  |  |  |  |  |  |  |  |  |
| TCM + Supportive care |  | × | × | × | × | × | × | × | × | × | × | × | × | × | × |  |  |
| Supportive care |  | × | × | × | × | × | × | × | × | × | × | × | × | × | × |  |  |
| **Baseline assessments** |  |  |  |  |  |  |  |  |  |  |  |  |  |  |  |  |  |
| Socio-demographics | × |  |  |  |  |  |  |  |  |  |  |  |  |  |  |  |  |
| Medical history | × |  |  |  |  |  |  |  |  |  |  |  |  |  |  |  |  |
| Differentiation | × |  |  |  |  |  |  |  |  |  |  |  |  |  |  |  |  |
| Routine inspection | × |  |  |  |  |  |  |  |  |  |  |  |  |  |  |  |  |
| Concomitant medication | × | × | × | × | × | × | × | × | × | × | × | × | × | × | × | × | × |
| **Outcome assessments** |  |  |  |  |  |  |  |  |  |  |  |  |  |  |  |  |  |
| Syndromes Relief | × |  |  | × |  |  |  | × |  |  | × |  |  |  | × |  |  |
| Coronavirus nucleic acid test | × |  |  | × |  |  |  | × |  |  | × |  |  |  | × |  |  |
| TCM Syndrome Scoring System | × |  |  | × |  |  |  | × |  |  | × |  |  |  | × | × | × |
| **Safety outcomes** |  |  |  |  |  |  |  |  |  |  |  |  |  |  |  |  |  |
| Routine inspection | × |  |  | × |  |  |  | × |  |  | × |  |  |  | × | × | × |
| Urine routine test | × |  |  | × |  |  |  | × |  |  | × |  |  |  | × | × | × |
| Blood rne test | × |  |  | × |  |  |  | × |  |  | × |  |  |  | × | × | × |
| Biochemical test | × |  |  | × |  |  |  | × |  |  | × |  |  |  | × | × | × |
| Electrocardiogram | × |  |  | × |  |  |  | × |  |  | × |  |  |  | × | × | × |
| Adverse events |  | × | × | × | × | × | × | × | × | × | × | × | × | × | × | × | × |

**Fig.2** SPIRIT schedule for enrollment,treatment and assessments.

D day,TCM Traditional Chinese Medicine
